# Energy-Saving Optimization Method of Urban Rail Transit Based on Improved Differential Evolution Algorithm

**DOI:** 10.3390/s23010378

**Published:** 2022-12-29

**Authors:** Guancheng Lu, Deqiang He, Jinlai Zhang

**Affiliations:** 1 School of Mechanical and Automotive Engineering, South China University of Technology, Guangzhou 510640, China; 2 School of Mechanical Engineering, Guangxi University, Nanning 530004, China

**Keywords:** urban rail transit, energy-saving optimization, adaptive dynamic multimodal differential evolution algorithm

## Abstract

The transformation of railway infrastructure and traction equipment is an ideal way to realize energy savings of urban rail transit trains. However, upgrading railway infrastructure and traction equipment is a high investment and difficult process. To produce energy-savings in the urban rail transit system without changing the existing infrastructure, we propose an energy-saving optimization method by optimizing the traction curve of the train. Firstly, after analyzing the relationship between the idle distance and running energy-savings, an optimization method of traction energy-savings based on the combination of the inertia motion and energy optimization is established by taking the maximum idle distance as the objective; and the maximum allowable running speed, passenger comfort, train timetable, maximum allowable acceleration and kinematics equation as constraints. Secondly, a solution method based on the combination of the adaptive dynamic multimodal differential evolution algorithm and the Q learning algorithm is applied to solve the optimization model of energy-savings. Finally, numeric experiments are conducted to verify the proposed method. Extensive experiments demonstrate the effectiveness of the proposed method. The results show that the method has significant energy-saving properties, saving energy by about 11.2%.

## 1. Introduction

To ensure the low-cost of urban rail transportation, energy-saving has become a key issue of the urban rail transportation system [1]. Although improving equipment and infrastructure reduces the operation cost of urban rail transit, these improvements require much more time and a higher investment. For this reason, exploring an optimization strategy to reduce the energy consumption of trains without changing the existing equipment and infrastructure has become an important research topic in recent years [2,3]. Due to the fact that the energy consumption of urban rail accounts for a large proportion of the public transport system, it is of great significance to realize energy savings in the urban rail [4,5], which will improve its own economic and social benefits. Based on the analysis of the existing research, a new traction energy-saving optimization based on the combination of train inertia motion and energy optimization is proposed in this study. The research purpose is to find a new energy-saving solution that reduces the energy conversion link as much as possible and replaces the braking motion with the inertia motion as much as possible.

At present, the research on energy-saving optimization of the traction is mainly focused on two aspects: traction energy-saving optimization based on regenerative braking energy utilization and traction energy-saving optimization based on the timetable. Some achievements have been obtained from these two research directions. They have their own advantages and disadvantages. The energy-saving optimization method of feeding regenerative braking energy into the recovery of the energy consumption optimization objective function still has a lot of energy loss in practical applications due to the influence of energy conversion efficiency and harmonic components. The practical application of the energy-saving optimization method by optimizing the timetable is not ideal due to the problem of condition simplification.

The energy-saving optimization based on the utilization of regenerative braking energy has been a hot research area in recent years. Typical research mainly includes the following aspects. Arikan and Yamur et al. adopted a combined strategy that includes reducing electrification loss, using regenerative braking, and improving comfort and driving technology to solve the problem of the high energy consumption of the train [6]. An optimization method for improving the energy efficiency of regenerative braking based on the master-slave mode is proposed [7]. On the basis of describing the switching model of urban rail train dynamics, an online optimization method of a train for energy-saving based on model predictive control is proposed [8]. To solve the problem that the energy consumption of a train is affected by speed control and the scheduled time, an energy-saving optimization method based on the flow conservation theory is proposed [9]. To reduce the energy consumption of the train, a multi-mode energy-saving rail transit simulator is established by matching the target speed, the average deceleration level and the adjustment coefficient of the braking force to match the precompiled time schedule of the train [10]. To achieve the goal of minimizing energy consumption, a train ecological driving strategy combined with a detailed train simulator is proposed by Cunillera and Alejandro, et al. [11]. A collaborative energy-saving optimization control strategy based on multi-agent deep reinforcement learning is proposed [12]. From the analysis of comprehensive energy-saving effects, these studies have achieved some results. However, the train energy-saving optimization method based on the comprehensive utilization of regenerative braking energy is restricted by energy storage equipment and harmonics; therefore, the effective utilization of regenerative braking energy is not high.

The representative research of the energy-saving optimization based on the train timetable includes the following topics. Comprehensively considering energy-saving and voltage-stabilization, an intelligent optimization strategy of energy-saving based on deep reinforcement learning is proposed [13]. An integrated model of the optimal train schedule and vehicle transfer plan is proposed by maximizing the overlapping the time of station braking and traction [14]. Shuo Zhao et al. construct the two-way ladder maximum interval function based on time-varying road demand and the predetermined service level [15]. A comprehensive optimization model of urban rail transit is proposed based on three sub-problems: line planning, timetable and vehicle allocation [16]. The essence of solving the train energy-saving optimization problem based on train operation schedule optimization is to transform the traction curve energy-saving optimization problem into the operation schedule optimization problem. Although the research in this field has achieved many results, there are still some problems to be solved. These problems to be solved mainly include that the constraints of the optimization objective function in the modeling process are too idealized, constraint conditions of the traction curve energy-saving model are too simplified and stay time of the station is not taking into account the economic benefits, and so on.

Although the previous research has achieved many results, there is still a lot of research space in the field of train energy-saving optimization. Without changing the existing infrastructure, the energy-saving method based on traction curve optimization is the key to achieving energy-savings. However, the optimization method of the traction curve for energy-saving is a multi-objective optimization problem that comprehensively considers the mutual restriction of operation safety, line infrastructure, traction conditions and operation energy-savings [17,18,19]. The interaction of multiple factors leads to the difference in train energy consumption in different environments and different operations, among which traction force, braking force, traction condition and braking condition have an obvious influence on the energy consumption of the train [18,20,21]. The idling based on the inertial motion of the train is an important operation mode to reduce energy consumption and provides the optimal conditions for energy-saving [21,22]. The time schedule of the train is given in advance and the given time is always greater than the minimum running time [22]. There are many train speed curves that meet the constraints such as time schedules. As far as the comprehensive benefit of urban rail transit is concerned, properly increasing the distance of the inertial motion within the allowable running time will effectively reduce the energy consumption of the train in the process of traction [23,24]. The traction energy-saving optimization of the train is a complex multimodal optimization problem. In practical application, due to the uncertainty of the problem environment or dynamic characteristics of the problem itself, the problem to be optimized will dynamically change with time or the environment [25,26,27]. The introduction of dynamic characteristics aggravates the complexity and difficulty of solving multimodal optimization problems, which poses a great challenge to the existing multimodal optimization algorithms [28,29].

From the previous research results, because of the excessive differences between modeling conditions and practical application conditions, the traction energy-saving optimization of the train based on the timetable does not solve the actual energy-saving optimization problem. There is energy conversion in the process of regenerative braking energy recovery. The optimization method based on the recovery and utilization of the regenerative braking energy depends on the conversion efficiency of the equipment and harmonic component of the regenerative braking energy. The energy conversion process is bound to reduce the actual effective utilization of energy due to the influence of equipment performance and energy conversion efficiency. From the view of energy conservation, the traction curve optimization method based on the optimization principle of regenerative braking energy utilization still has a lot of energy loss. The main reason for this problem is that the energy conversion efficiency is not completely converted into the actual effective use of energy. Therefore, reducing the unnecessary conversion and recovery of regenerative braking energy as much as possible is the key to realizing deep energy-saving optimization of the traction curve. The combination of the energy optimization and train inertia motion is an important research direction to realize the energy-saving optimization in the urban rail transit.

Traction energy-consumption is the major energy-consumption of the urban rail transit. The energy-saving optimization studies described herein are all aimed at traction energy-saving optimization research. Establishing a good traction curve to improve the train operation is an important way to produce energy-savings in the urban rail transit. To establish a good traction curve to realize energy savings by improving the train operation in the urban rail transit without changing the existing infrastructure, this study will research the traction energy-saving optimization method of the train based on the combination of inertia motion and energy optimization. We use inertia motion to replace the braking motion as much as possible by reasonably optimizing the operation curve of the train, so as to avoid various energy conversion links and effectively improve the actual energy efficiency. An optimization method of traction energy-saving based on the combination of inertia motion and energy optimization is proposed to solve the traction energy-saving optimization problem. The method studied is different from that of predecessors. The method proposed in this study is neither directly based on the regenerative braking energy feedback to the optimization objective function nor simply using the running schedule as the optimization objective function, but comprehensively considers the combination of the train inertia motion and energy optimization. The proposed method provides a new reference solution for the optimization problem of energy-saving in train operation. The main contributions of this study are summarized as follows:

(1) An optimization model based on the combination of the inertia motion and energy optimization is established by taking the maximum idle distance as the objective and the maximum allowable running speed, passenger comfort, train timetable, maximum allowable acceleration and kinematics equation as constraints.
(2) An improved differential evolution algorithm based on the adaptive dynamic multimodal model is proposed and a comprehensive method for solving multi-objective optimization problems based on the combination of the improved differential evolution algorithm and Q learning algorithm is applied to solve the energy-saving optimization model.

## 2. Methods

The harmonic component of regenerative braking energy, performance of the energy recovery equipment and energy conversion efficiency are the key factors of the traction energy-saving optimization method based on the regenerative braking energy utilization. These key factors result in the regenerative braking energy not fully converting into the actual effective energy and there is much waste energy. The research method in this study is different from most of the previous research methods and does not rely solely on the utilization of regenerative braking energy and the train operation schedule. To find a new traction energy-saving method based on the combination of energy optimization and the inertial motion of the train is the main purpose of the study. The kinematic analysis and calculation of the energy-consumption is the key to establishing the traction energy-saving model of the train. The modeling principle and construction method of the optimization objective function are the core of the traction energy-saving optimization problem. In this section, we will describe the energy-saving optimization method proposed in this study in detail. Section 2.1 is the modeling basis including the calculation of the running resistance and kinematic analysis of the train. Section 2.2 is the modeling principle and the specific modeling implementation of the traction energy-saving optimization problem. Section 2.3 is the solution method and specific implementation of the energy-saving optimization model.

### 2.1. Basis

#### 2.1.1. Calculation of the Running Resistance

Because the operation of the train is affected by many factors and there are many complex relationships between the factors, it is very difficult to use an accurate mathematical model and calculation formula to obtain the running resistance of the train. The calculation formula based on the empirical formula is the main means to obtain the actual running resistance of the train. According to the empirical formula, the basic unit motion resistance of the train is expressed by Formula (1).

(1) $$w_{b}=c_{1}\cdot v^{2}+c_{2}\cdot v+c_{3}$$

The additional resistance of the train is the external resistance exerted by the line when the train runs on it. The additional resistance of the train is mainly related to the line conditions. The additional resistances of the train mainly include the tunnel additional resistance, curve additional resistance, ramp additional resistance and so on. The generation of the tunnel additional resistance is due to the fact that the fast-running train reduces the cross-sectional area of airflow and produces resistance that hinders the movement of the train under the aerodynamic effect. The unit additional resistance of the tunnel can be calculated from the empirical formula shown in Formula (2).

(2) $$w_{c}=\left\{ \begin{array}{ll} L\cdot v^{2}\cdot 10^{-7} & ,\mathrm{arc}\;\mathrm{tunnel}\\ 0.00013L & ,\mathrm{line}\;\mathrm{tunnel} \end{array}\right.$$

When the train is running on the curved track, the curved track will drag the train and cause additional resistance in the curved track. Due to the complexity of influencing factors, it is very difficult to calculate the additional resistance by theoretical calculation. The additional resistance of the curved track is usually calculated by train weight and unit additional resistance, and it can be calculated by the empirical formula shown in Formula (3). In Equation (3), *w*_r_ denotes the unit additional resistance of the curved track, and its unit is N/kN; *c*_4_ is an empirical constant; *R* denotes the radius of the curved track, and its unit is m.

(3) $$w_{r}=\frac{c_{4}}{R}$$

Due to the action of gravity, the train is hindered by the resistance along the inclined track on the ramp. The unit additional resistance of the ramp is usually calculated by the slope thousands of points, which is equal to the thousand fractions of the ramp. The symbol *w*_p_ is used to represent the unit additional resistance of the ramp. In the case of considering the basic resistance, the additional resistance of the tunnel, additional resistance of the curved track and additional resistance of the ramp, the instantaneous motion resistance of the train can be calculated by Formula (4).

(4) $$W=(w_{b}+w_{c}+w_{r}+w_{p})\cdot (P+G)\cdot g\cdot 10^{-3}$$

To analyze the train kinematics, the distance between the two stations is divided into many small segments. Assuming that the distance between the two stations is *S*, the length of each segment is calculated by Formula (5). When the segment is small enough, the unit resistance of the train in the segment is approximately calculated by the average speed of the train and empirical formula. The instantaneous motion resistance of the train can be approximated by the average motion resistance of the train when the segment is small enough. The average unit motion resistance and average motion resistance of the train in the small segment can be calculated by Formulas (6) and (7), respectively.

(5) $$\Delta s_{i}=\frac{S}{n}(i=1,2,\ldots ,n)$$

(6) $$\bar{w_{b}}=c_{1}\cdot {\bar{v_{i}}}^{2}+c_{2}\cdot \bar{v_{i}}+c_{3}$$

(7) $$\bar{W}=(\bar{w_{b}}+w_{c}+w_{r}+w_{p})\cdot (P+G)\cdot g\cdot 10^{-3}$$

#### 2.1.2. Kinematic Analysis

The kinetic energy of the train is generally composed of translational kinetic energy and rotational kinetic energy. After introducing the rotary mass coefficient, the kinetic energy of the train is calculated by Formula (8). According to the derivation of the train kinetic energy calculation and principle that the change in energy consumption can be represented by the increment of the kinetic energy, the kinetic energy increment of the train can be expressed by Formula (9).

(8) $$E_{k}=\frac{Mv^{2}}{2\cdot 3.6^{2}}(1+p)$$

(9) $$dE_{k}=Mv(1+p)dv$$

To obtain the relationship between the increment of the kinetic energy and work done by the external resultant force, the running time of the train is represented by *t* in s, running distance of the train is represented by s in m, and external resultant force on the train is represented by C in kN. According to the principle that the work done by the external force acting on the object is equal to the change in the kinetic energy of the object, that is Δ*E*_k_ = *Cs*, the equation of motion for the train can be described by Equation (10). After Formula (10) is rearranged, the train running time equation and running distance equation can be expressed by Formulas (11) and (12).

(10) Mv(1+p)dv=3.6Cvdt

(11) $$dt=\int \frac{M(1+p)}{3.6C}dv$$

(12) $$ds=\int \frac{Mv(1+p)}{3.6^{2}C}dv$$

When the distance between the two stations is discretized into sufficient small segments, the kinematic equations of the train on the small segments can be described by Equations (13) and (14).

(13) $$\Delta t_{i}=\frac{M(1+p)(v_{ie}-v_{is})}{3.6C_{i}}$$

(14) $$\Delta s_{i}=\frac{M(1+p)(v_{is}+v_{ie})(v_{ie}-v_{is})}{2\cdot 3.6^{2}C_{i}}$$

### 2.2. Modeling

#### 2.2.1. Modeling Principle

The external forces closely related to the energy consumption of train operation mainly include traction force, basic resistance, additional resistance and braking force. In the urban rail transit system, the external resultant force on the train determines the running state of the whole train. The operation of the train generally includes the traction condition, cruising condition, inert condition and braking condition. When the train is running in the traction condition, the external force on the train mainly includes locomotive traction and motion resistance, and the external resultant force acting on the train is equal to the traction force, subtracting the motion resistance. When the train is running in the idling condition, the train is only affected by the motion resistance, and the external resultant force is equal to the opposite number of the motion resistance, and it does not consume electric energy but depends on inertia to slow down. When the train is running in the cruising condition, the resistance of the train is equal to the traction force, the external resultant force acting on the train is equal to 0, and the train consumes energy and moves forward at a uniform speed. When the train is running in the braking condition, the train is affected by the braking force and motion resistance, the traction motor enters the power generation mode, and the train produces regenerative braking energy and slows down. The interaction of multiple factors leads to the difference in train energy consumption in different working conditions, among which traction force, braking force, traction condition and braking condition have obvious influence on the energy consumption of the train. From the view of traction energy consumption the idling mode, which only depends on the inertia to glide forward, is an important energy-saving operation.

At present, the research on the optimization of traction energy-saving based on the utilization of the regenerative braking energy and optimization of traction energy-saving based on the train timetable are the focus research directions of the traction energy-saving optimization. However, the utilization of regenerative braking energy is easily affected by harmonic components and energy conversion efficiency, and the overall energy still produces a lot of waste. The research on the optimization of traction energy-saving based on the train timetable mainly improves the energy consumption of the train by optimizing the train timetable. However, it mainly focuses on energy savings from the aspect of dispatching management. It does not study the solution curve for the energy-saving optimization problem from the aspects of velocity curve and acceleration curve. Based on the combination of energy optimization and train inertia motion, traction energy-saving optimization is studied, which provides a new research scheme for the traction energy-saving optimization of the train. 

The process of train operation is a process that contains a variety of complex forms of motion. The energy conversion mechanism of the train operation process is so complex that it is difficult to reveal the full energy conversion mechanism through a mathematical expression. The energy-saving optimization problem of the traction is a complex optimization problem with optimization constraints but no clear mathematical analytical optimization law. At present, a clear mathematical description of the optimization problem has not been found. Most of the optimization objective functions are established according to the actual optimization conditions. When establishing the energy-saving optimization objective function, this study still adopts the previous research method, that is, to construct the optimization objective function through the optimization conditions.

**Figure 1:**
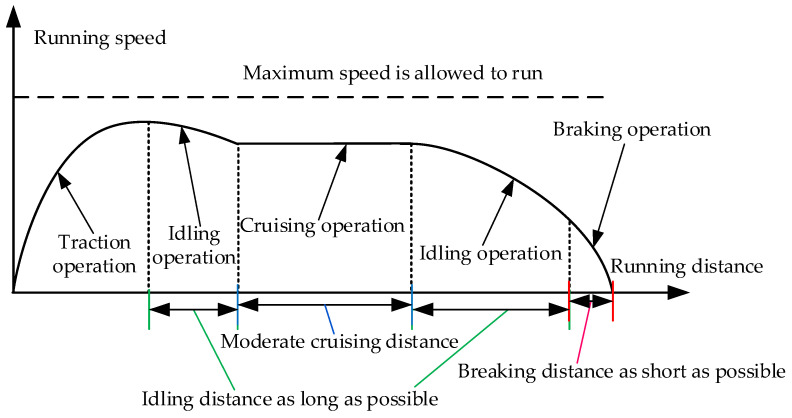
Schematic diagram of maximum idling distance optimization.

The basic principle of optimization of traction energy-saving is that the total energy consumption of traction is as small as possible, running distance of the train is as long as possible, and train arrives on time as far as possible. Under the condition that the arrival time allows a small range of fluctuations, the replacement of the distance of the regenerative braking by the idling distance not only effectively saves the energy of the train over the whole distance between the two stations, but also shortens the distance of regenerative braking. The time schedule of the train to finish the whole distance between the two stations is always given in advance, and the given time is always greater than the minimum running time, so there are many speed curves that meet such a running time. The increase in idling distance appropriately reduces the braking distance, and solves the problem of low recovery and utilization of the regenerative braking energy. The schematic diagram of the maximum idling distance for the energy-saving of the train is shown in Figure 1.

#### 2.2.2. Modeling Implementation

The energy-saving optimization studied in this study is to find a solution on the basis of meeting the requirements of basic operation regulations, which makes the train run the furthest distance depending on inertia motion. Punctual departure, punctual arrival, accurate parking and improving passenger comfort as much as possible are the basic operation requirements of the urban rail train. Punctual departure and punctual arrival require that the train must be completed within the specified time when it runs from one station to another. The actual running time between the two stations allows a slight deviation from the specified time. Urban rail transit is always equipped with safety screen doors to ensure the safety of passengers. The train must accurately park at the safety screen door; otherwise, because of the dislocation between the train door and safety screen door, passengers will be unable to get on and off the train. Accurate parking requires that the final speed of the train must be 0 when it reaches the target position of the safety screen door. To improve passenger comfort as much as possible requires that the acceleration be considered in the process of train operation, acceleration must be within the prescribed range, and gradient between the two adjacent accelerations must meet the passenger comfort factor. Additionally, the train traction is not inexhaustible, but there is an upper limit. Because in the process of optimization, it is necessary to calculate the actual traction force and judge whether it is within a reasonable range. If the optimization process finds that the tractive force is not within a reasonable range, the corresponding solution sequence must be punished. Therefore, the train energy-saving optimization problem studied is actually a multi-objective optimization problem composed of the objective of running time, inertial motion, change of adjacent accelerations and final speed at the destination station.

Because the environment of operation in the urban rail transit is often different and the deviation of allowable running time of the train between stations is always different, the traction scheme of the train is different. The reasonable conversion scheme of idling working condition is an important guarantee for the energy-saving of the train. Therefore, an objective function of the maximum idling distance of the train is constructed in the study based on the natural exponential function, as shown in Formula (15). The monotonicity of the natural exponential function and its range from 0 to 1 are used to express the optimization objective of the idling distance. When the idling distance of the train becomes larger, the value of the objective function becomes smaller, and vice versa.

(15) $$\min F_{1}=e^{-\sum_{k=1}^{n}\Delta s_{k}}$$

The requirements of the operation schedule for the train are mainly reflected in the punctual arrival. The punctual arrival of the train is generally characterized by the punctual rate of the train. The punctual rate of the train not only affects the safety of the urban rail transit in the process of operation, but also directly restricts the optimization scheme of the energy consumption for the train. According to the time schedule of the train, the arrival time of the train allows a small range of fluctuations under the basic requirements of the given operation schedule, which provides a running time optimization condition for the energy-saving traction of the train. Therefore, the total running time of the train approaching the time schedule is a sub-goal of the energy-saving optimization problem under the constraint of the operation schedule for the train. When the time schedule of the train between the two stations is represented by *T*, an objective function of the optimization for the punctual arrival of the train is constructed, which is shown as in Formula (16).

(16) $$\min F_{2}={\left(\sum_{i=1}^{N}\Delta t_{i}-T\right)}^{2}$$

The change in train acceleration is an important factor affecting the comfort of train passengers. If the train acceleration changes slowly, passengers get high comfort; the faster the change in the train acceleration, the more passengers will feel uncomfortable. In the energy-saving optimization problem of the train, it is bound to control the speed and acceleration of the train traction curve, which will inevitably lead to the change in train acceleration and ultimately affect the comfort of passengers. Therefore, an optimization objective function of passenger comfort is constructed based on the change rate of the acceleration. It is shown as in Formula (17).

(17) $$\min F_{3}={\left(a_{i+1}-a_{i}-\vartheta \right)}^{2}$$

In the whole optimization problem of energy-saving, the final speed of the train is also a part of the energy-saving problem when the train arrives at the destination station. It means that the speed of the train must be close to zero when the train arrives at the target station after the speed of the train changes in the series of operation conditions. For this reason, the final speed optimization objective function is constructed, which is shown as in Formula (18). The symbol *v_Ne_* in Formula (18) represents the final speed of the train when it arrives at the target station.

(18) $$\min F_{4}={\left(v_{Ne}-0\right)}^{2}$$

After the sub-optimization problems to form the overall optimization objective function is organically combined, the whole energy-saving optimization model of the train is shown in Formula (19).

For multi-objective optimization problems such as train energy-saving optimization, the more complete the conditions considered, the better the optimization effect obtained, but the more complex the solution process becomes. This study explores the optimization method of train energy savings based on inertia motion under the requirements of the basic operating regulations of the urban rail train. Therefore, to meet the requirements of the basic operation regulations and obtain the solution of the energy-saving optimization is the main goal of the multi-objective optimization in this study. In the energy-saving multi-objective optimization function, several sub-optimization objectives are the key objectives, none of which are indispensable. If there is a lack of the optimization objective of the final speed of the train, the optimization results may result in a running distance to the position of the safety screen door but the train speed is not zero, resulting in the dislocation of the train door and the safety screen door. If there is a lack of the optimization objective for the change in the adjacent accelerations, then the optimization results may result in a sudden jump in the acceleration, which seriously affects the comfort of passengers. If the optimization objective of running time is lacking, then the optimization results may have a conflict with the running timetable, resulting in the safety problem of the train scheduling.

(19) $$\begin{array}{l} \min\Phi =\alpha {\left(\sum_{i=1}^{n}\Delta t_{i}-T\right)}^{2}+\beta {\left(v_{ne}-0\right)}^{2}+\gamma e^{-\sum_{k=1}^{n}\Delta s_{k}}+\lambda {\left(a_{i+1}-a_{i}-\xi \right)}^{2}\\ s.t.\left\{ \begin{array}{l} \Delta s_{i}=\frac{S}{n}\\ \Delta s_{k}=\left\{ \begin{array}{l} \Delta s_{i},v_{ie}<v_{is}\\ 0,v_{ie}\geq v_{is} \end{array}\right.\\ a_{i}=\frac{C_{i}}{\left(P+F_{ci}G\right)}\\ \Delta t_{i}=\left\{ \begin{array}{l} \frac{\left(P+F_{ci}G)(1+p)(v_{ie}-v_{is}\right)}{3.6C_{i}},v_{ie}\neq v_{is}\\ \frac{\Delta s_{i}}{v_{ie}},v_{ie}=v_{is}\;\mathrm{and}\;v_{ie}\neq 0\\ 0,\mathrm{others}\; \end{array}\right.\\ C_{i}=\frac{\left(P+F_{ci}G)(1+p)(v_{is}+v_{ie})(v_{ie}-v_{is}\right)}{2\cdot 3.6^{2}\Delta s_{i}}\\ \bar{W_{i}}=\frac{\left[c_{1}{\left(\frac{v_{ie}+v_{is}}{2}\right)}^{2}+c_{2}\frac{v_{ie}+v_{is}}{2}+c_{3}+w_{c}+w_{r}+w_{p}\right]}{1000}(P+F_{ci}G)g\\ F_{Ti}=\left\{ \begin{array}{l} C_{i}-\bar{W_{i}},v_{ie}\geq v_{is}\\ 0,v_{ie}<v_{is} \end{array}\right.\\ 0\leq v_{is}\leq v_{\max}\\ 0\leq v_{ie}\leq v_{\max}\\ 0\leq F_{Ti}\leq F_{T\max}\\ \left|a_{i}\right|\leq \left|a_{\max}\right|\\ v_{1s}=0 \end{array}\right. \end{array}$$

### 2.3. Solution

The energy-saving optimization problem of urban rail transit is not only a complex optimization problem with a high density of switching points and high computational complexity, but also a multimodal optimization problem closely related to the evolution of the operating environment. Due to the uncertainty and dynamic characteristics brought by the evolution of the urban rail transit operation environment, the solution of the urban rail transit energy-saving optimization problem presents the characteristics of multi-mode and dynamic coexistence. The superposition of dynamic and multi-mode on the optimization problem aggravates the complexity and difficulty of solving the optimization problem. The classical numerical solution of multi-objective optimization problems and ordinary swarm intelligence algorithms are difficult to adapt to this kind of problem, which makes it difficult to obtain satisfactory solutions or even fail under limited computational conditions. To solve the optimization model of the traction energy-saving, the adaptive dynamic multimodal differential evolution algorithm is introduced into the urban rail transit energy-saving optimization problem and a solution method based on the improved differential evolution algorithm is used to solve the optimization model.

#### 2.3.1. Improved Differential Evolution Algorithm Based on the Adaptive Dynamic Multimodal Model

The basic principle of the standard differential evolution algorithm is referred to in [25,28]. In this study, we mainly improve the standard differential evolution algorithm based on the adaptive perception mechanism of the best fitness gradient and dynamic multimodal mechanism of the unsolved optimization problem, obtain an adaptive dynamic multimodal differential evolution algorithm, and apply it to the solving process of the urban rail transit energy-saving optimization problem. The individual adaptive hierarchical mutation and adaptive knowledge learning in the adaptive dynamic multimodal differential evolution algorithm are realized by the comprehensive learning of population global knowledge and individual neighborhood knowledge. Finally, a satisfactory solution to the energy-saving optimization problem of urban rail transit is obtained by the dynamic adaptive response mechanism, global exploration balance mechanism and local neighborhood knowledge comprehensive learning mechanism.

Affected by the dynamic characteristics, quickly determining the possible position of the optimal value is an important way to improve the convergence speed and diversity of solving the multi-objective optimization problems with dynamic and multimodal co-existence. Therefore, how to make full use of the knowledge of the population evolution process to guide the population to achieve fast and accurate convergence within a limited number of evaluations is the key to solving the dynamic multimodal optimization problems with high quality. However, the traditional differential evolution algorithm, which generates offspring only through a simple mutation crossover operator, does not meet the dual requirements of convergence speed and diversity in the solving process of dynamic multimodal optimization problems. Therefore, this study introduces the knowledge guidance mechanism into the differential evolution algorithm to construct an adaptive difference algorithm based on the knowledge guidance mechanism. The main principle is to extract the information of the current evolutionary state of the population and guide individuals to adaptively select the variation that best matches the current evolutionary state according to the evolutionary needs of different individuals. The main steps of the method are described below.

(1) Acquire the knowledge of the population evolution process and judge the individual evolution needs of the population.
(2) Construct the population diversity enhancement strategy to help the population jump out of the stagnant region.
(3) According to the evolution state of the population and individual, the hierarchical mutation method is used to adaptively select the most suitable variation mode of the current individual, and guide the individual to learn knowledge from the global evolution information and neighborhood information of the population.

In this study, the degree of change of the optimal fitness value of each generation in the process of evolution is taken as a reference to extract the evolutionary state of the current population. The formula for calculating the degree of change of the optimal fitness value of the population is shown in Formula (20).

(20) $$\theta =\frac{\left|F_{best}(k)-F_{best}(k-1)\right|}{2}$$

The diversity enhancement strategy mechanism adopted in this study is to guide other individuals of the population to jump out of the current local region and move to other unexplored areas on the basis of retaining multiple peak optimal solutions currently found, so as to improve group diversity and exploration ability. The specific enhancement process is shown in the following Formula (21). Where *x_ir1_*, *x_ir2_*, *x_ir3_*, *x_ir4_* and *x_ir5_* represents three different random values of the *i* individual, respectively.

(21) $$x_{ij}=\left\{ \begin{array}{l} x_{ij},\mathrm{when}\;\mathrm{rand}(0,1)<CM\\ x_{ir1}+SC(x_{ir2}-x_{ir3})+\theta (x_{ir4}-x_{ir5}),x_{ir1}\neq x_{ir2}\neq x_{ir3}\neq x_{ir4}\neq x_{ir5},\mathrm{others} \end{array}\right.$$

In this study, based on the elite individual strategy, the convergence mechanism of the search is constructed, and the global convergence of the solution of the optimization problem is realized by randomly exchanging a certain dimension value of the most individuals to the optimal solution. The update formula of the convergence mechanism is shown in Formula (22). Where *x_ij_* represents the *j* dimension of the *i* individual and *rand* (0,1) represents a random number from 0 to 1.

(22) $$x_{ij}=\left\{ \begin{array}{l} x_{bestj},\mathrm{when}\;rand(0,1)<CR\\ x_{ij},\mathrm{others} \end{array}\right.$$

To improve the adaptive ability of the differential evolution algorithm and ensure that the algorithm can quickly obtain a satisfactory solution to the energy-saving optimization problem of urban rail transit within limited computing resources, an adaptive hierarchical mutation strategy of erasing the evolutionary algorithm is constructed based on the knowledge guidance mechanism through the comprehensive learning of population evolution process knowledge. The individuals of different evolutionary states are guided to adaptively choose the best way of variation to meet the evolutionary needs of different individuals. The main implementation steps are as follows.

(1) Set the threshold *δ* of the change degree of the optimal adaptation value of the population.
(2) If the degree of change of the optimal fitness value of each generation *θ* is *δ*, it means that the population may still be rapidly approaching the optimal solution, and the convergence of the population is improved at this time.
(3) If the degree of change of the optimal fitness value of each generation *θ* < *δ*, it means that the degree of evolution of the current population search is small, and the diversity of the population is enhanced at this time.

#### 2.3.2. Solution Implementation of the Optimization Model

According to the characteristics of the energy-saving optimization model and adaptive dynamic multimodal differential evolution algorithm, a method based on the penalty strategy of violating the constraint conditions is proposed to solve the optimization model in the study. A cost function to calculate the fitness of the individual in the adaptive dynamic multimodal differential evolution algorithm is constructed, which is shown as in Formula (23).

In the process of evaluating the solution of the optimization problem, the penalty strategies based on violation of constraints mainly include the traction acceleration process, and violation of the maximum speed constraint and passenger comfort constraint. The specific implementation process of increasing the penalty in violation of the maximum speed constraint is to give a large coefficient to the total energy consumption of the train when the small segment acceleration is greater than the maximum acceleration allowed to run. The specific implementation process of increasing the penalty in violation of the maximum speed constraint is to give a large coefficient to the total energy consumption of the train when the end speed of the small segment is greater than the maximum speed allowed. The specific implementation process of increasing the penalty against the passenger comfort constraint is that when the acceleration variation of the two adjacent segments is greater than the passenger comfort control factor, it produces a large coefficient to the total energy consumption of the train operation.

(23) $$F=\frac{1}{(\prod_{i=1}^{N}Z_{i})\cdot (\prod_{i=1}^{N}A_{i})\cdot (\prod_{i=1}^{N}V_{i})(\prod_{i=1}^{N}T_{i})\cdot f_{b}+\varepsilon }$$

(24) $$f_{b}=\alpha \cdot {\left(\frac{\sum_{i=1}^{N}\Delta t_{i}}{T}-1\right)}^{2}+\beta \cdot {\left(\frac{v_{Ne}}{v_{\max}}-0\right)}^{2}+\gamma \cdot e^{\sum_{k=1}^{N}\Delta s_{k}-s}+\tau \cdot \frac{{\left(a_{i+1}-a_{i}-\xi \right)}^{2}}{2\xi }$$

(25) $$Z_{i}=\left\{ \begin{array}{ll} \upsilon & ,\left|a_{i}\right|>\left|a_{\max}\right|\\ 1 & ,\mathrm{others} \end{array}\right.$$

(26) $$V_{i}=\left\{ \begin{array}{ll} \delta & ,\left|v_{is}\right|>\left|v_{\max}\right|\mathrm{or}\left|v_{ie}\right|>\left|v_{\max}\right|\\ 1 & ,\mathrm{others} \end{array}\right.$$

(27) $$A_{i}=\left\{ \begin{array}{ll} \frac{\xi }{\left|a_{i+1}-a_{i}\right|} & ,\left|a_{i+1}-a_{i}\right|>\xi \\ 1 & ,\mathrm{others} \end{array}\right.$$

(28) $$T_{i}=\left\{ \begin{array}{ll} \frac{1}{F_{T\max}-F_{Ti}} & ,F_{Ti}>F_{T\max}\\ 1 & ,F_{Ti}\leq F_{T\max} \end{array}\right.$$

In the multi-objective optimization problem, the solution that satisfies all the constraints at the same time is called a feasible solution, and the space composed of all feasible solutions is called the feasible region. When solving a multi-objective optimization problem, the ideal solution is that the optimal solution of the multi-objective problem satisfies the optimal solution of each objective at the same time. However, it is often difficult to achieve this goal because of the existing multi-objective functions. The optimal solution of the objective function is usually a set of solutions obtained by balancing each objective, which includes a feasible solution, Pareto domination solution and Pareto optimal solution. Because of the diversity of solutions of multi-objective optimization problems and most of the solutions that meet the constraints appear in the form of a combination, constructing a decision-making scheme of a satisfactory solution based on the actual meaning of the problem is a main method of eclectic solution for the multi-objective optimization problem.

There are a large number of non-inferior solutions in the direct optimization results of traction energy-saving optimization problems. The acquisition of a satisfactory solution to the problem of train traction energy-saving optimization is a key link in the implementation process of train traction energy-saving optimization. Because the train traction energy-saving optimization problem attaches different importance to each sub-optimization goal, it leads to the difference in the eclectic degree of the traction energy-saving parameter sequence to each sub-optimization problem. The energy-saving benefit of the optimization results, eclectic degree of sub-optimization problems and implementation of practical problems have become an important reference for the selection strategy of eclectic solution.

The Q learning algorithm is a classical reinforcement learning algorithm. Its principle is to take the agent as the core, state transition as the foundation, benefit as the goal, action reward value as the guidance; and based on the principle of execution and return, construct the action reward value matrix of the optimization problem by the form of iterating and updating [30]. Finally, the action reward value can be selected to obtain the maximum benefit. To obtain the best solution of the optimization problem, the Q learning algorithm does not depend on any model, and its agent can obtain the best path and action sequence from the initial state to the target state through learning in an unknown environment [31].

To obtain a satisfactory solution from the eclectic solution set, this study takes the Q learning algorithm as the core and the non-inferior solution posterior evaluation and optimization goal as the decision strategy to solve the problem of non-dominant solution choice. The intelligent perceptual recursive decision maker of the non-dominant solution set of the optimization problem is constructed based on the Q-learning algorithm; the identification process of the non-dominant solution set is transformed into a process of parameter transfer in different positions; and the elimination method is used to recursively iterate the non-dominant solution. Finally, a satisfactory solution of the traction energy-saving optimization problem is obtained.

**Figure 2:**
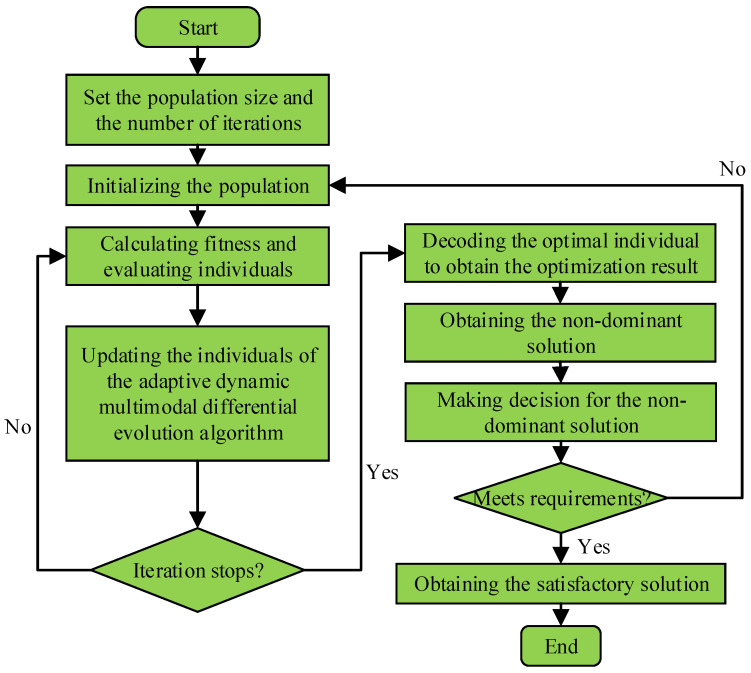
Overview of energy-saving optimization model.

A method combined with the adaptive dynamic multimodal differential evolution algorithm, the Q learning algorithm and hybrid coding algorithm is applied to solve the optimization problem in the study. The pipeline for solving the optimization problem is shown in Figure 2. The main process of solving the problem is as follows:

(1) Setting the population size and number of iterations;
(2) Initializing the population of the adaptive dynamic multimodal differential evolution algorithm;
(3) Calculating fitness and evaluating individuals of the adaptive dynamic multimodal differential evolution algorithm;
(4) Updating the individuals of the adaptive dynamic multimodal differential evolution algorithm;
(5) Determining whether the stop condition of the iteration is satisfied; if so, then turns to (6), and otherwise turns to (3);
(6) Decoding the optimal individual to obtain the optimization result;
(7) Obtaining the non-dominant solution of the optimization problem;
(8) Making a decision for the non-dominant solution by the Q learning algorithm;
(9) Whether the dominant solution meets the requirements of the problem; if so, then turns to (10), and otherwise turns to (2);
(10) Obtaining the satisfactory solution of the optimization problem.

## 3. Numeric Experiments

### 3.1. Experiment Setup

The historical operation data of the urban rail transits in Nanning, China is used as the basic comparative data. The simulation experiment and analysis of the proposed method are carried out based on MATLAB software and running parameters from the urban rail transits in Nanning, China. The main parameters of the simulation analysis are shown in Table 1. In the experiment, the distance between the two stations is 2200 m, upper limit speed is 80 km/h, maximum allowable running time is 180 s, average historical energy consumption of the train is 10,499 KJ, and actual average running time of the historical operation is 168 s.

The energy-saving method based on timetable optimization and energy-saving optimization method based on regenerative braking energy utilization are important research directions of train energy-saving optimization at present. To verify the advantages of the proposed methods in the overall energy-saving effect, three representative methods of these methods are selected for numerical experimental comparison. Taking the chicken swarm optimization algorithm as the solution method, this study uses the method proposed in reference [32] to compare the numerical experiments, and call it the method based on GA for short. The method is proposed to find the optimal operation curve and produce energy savings of the urban rail train based on a multi-objective improved genetic algorithm. Taking the chicken swarm optimization algorithm as the solution method, this study uses the method proposed in reference [33] for numerical experiment comparison, which is referred to as the method based on CSO for short. The method is proposed to obtain an operation curve of a train with minimum energy-consumption and improve the utilization of regenerative braking energy without changing equipment and infrastructure based on the improved chicken swarm optimization. Taking the particle swarm optimization algorithm as the solution method, this study uses the numerical experiment comparison of the method proposed in reference [34], which is referred to as the method based on PSO for short. The method is proposed to optimize the operation strategy together with the integrated train timetable and obtain the energy-efficient operation base on the particle swarm optimization. The methods used for the comparison of numerical experiments can be described in detail in the corresponding reference. 

In this study, the corresponding program is compiled according to the principle of the method used for comparison, and numerical experiments are carried out based on the urban rail transits in Nanning, China as the basic data of these methods. By taking the historical manipulation data as the basic comparison data, numeric experiments based on the different energy-saving optimization methods are carried out to compare and analyze the running energy consumption, running time and change in tractive force etc.

### 3.2. Experiment Results

**Figure 3:**
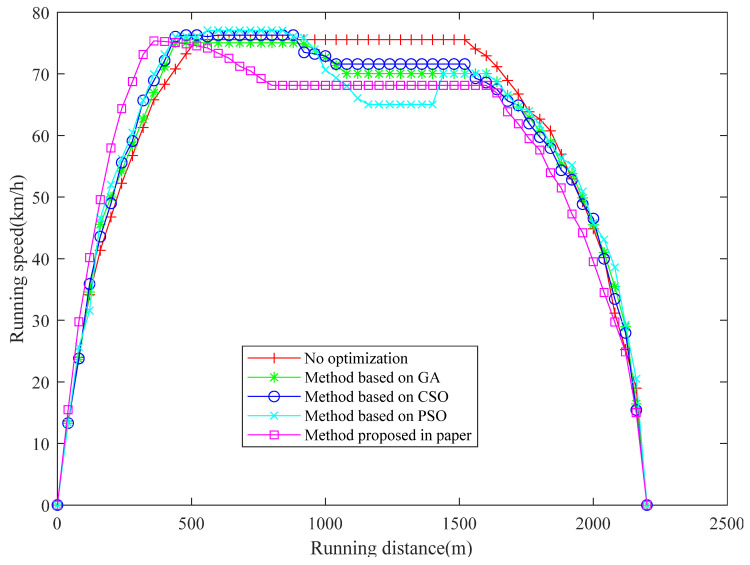
Running distance and running speed.

**Figure 4:**
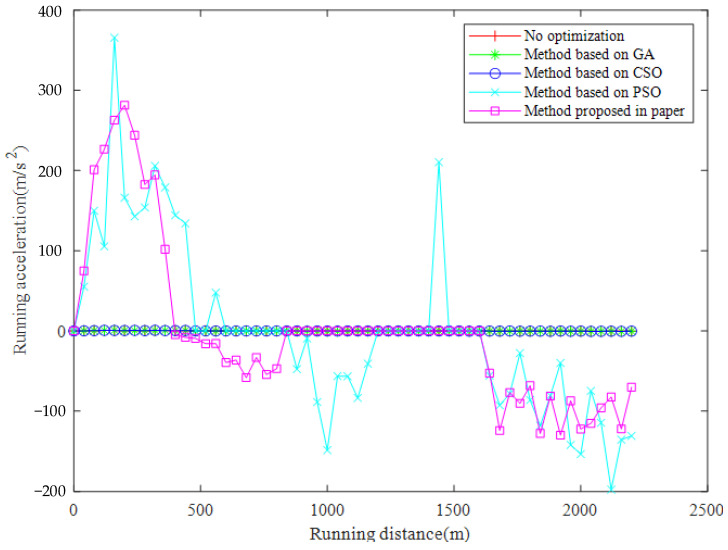
Running distance and running acceleration.

**Figure 5:**
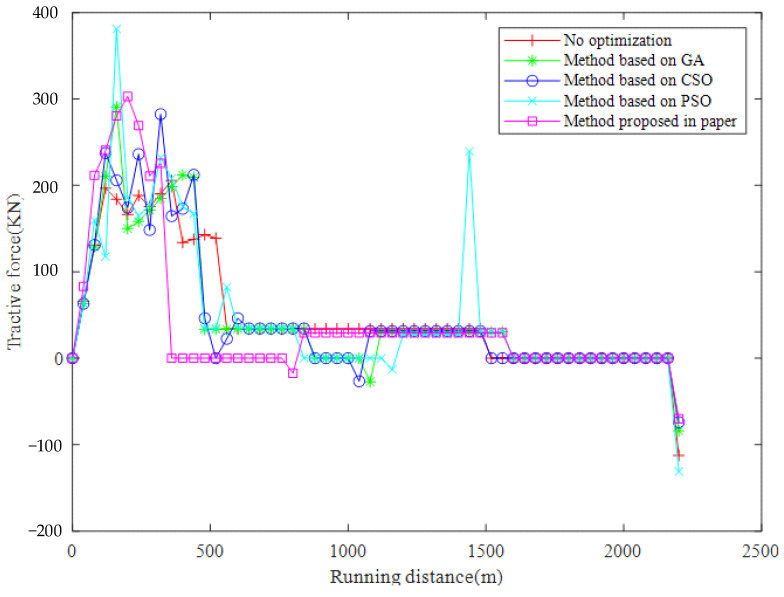
Running distance and tractive force.

**Figure 6:**
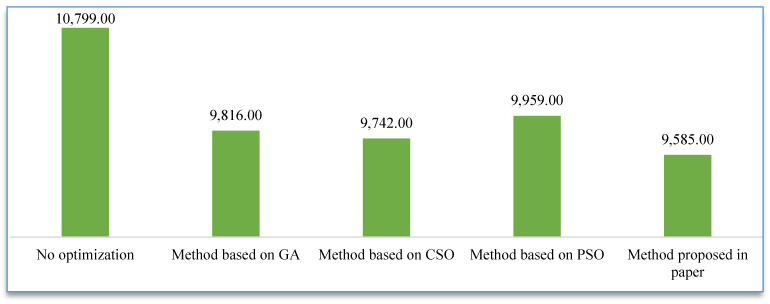
Comparison of energy consumption of different methods.

**Figure 7:**
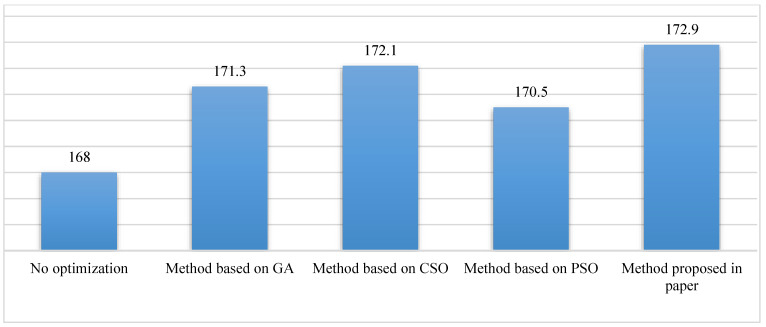
Comparison of run time of different methods.

The results of the numeric experiments are shown in Figure 3, Figure 4, Figure 5, Figure 6 and Figure 7, respectively. The speed curve of each optimization method conducted in the numeric experiments is shown in Figure 3. The Figure shows that the speed curve of each method is within the maximum speed limit, and the value is reasonable. The acceleration curve for each optimization method is shown in Figure 4. Figure 4 shows that the acceleration curve of each method is within the maximum limit value. Figure 5 shows that the tractive force of each optimization method is less than the maximum tractive force, which is 330 kN. The energy consumption of each optimization method is shown in Figure 6. The results in Figure 6 show that the energy consumption of the proposed method is the smallest. It shows that the traction energy consumption is significantly reduced under the same running distance after the operation is optimized by the proposed method. The running time of each optimization method is shown in Figure 7. From the numerical experimental results shown in Figure 7, the running time of the method proposed is the largest. However, from the numerical experimental results, the running time of several optimization methods is not very different, and it is acceptable in the actual operation.

### 3.3. Experimental Evaluation

The results of the experiment show that the proposed method is effective. Although it takes a little longer to run the whole distance, it saves much more energy than other methods in the experiment. From the overall energy-saving point of view, compared with the historical operation data, the proposed method saves energy by about 11.2%, while the other optimization methods save energy by about 9.1%, 9.7%, and 7.7% respectively. Although the purpose of these optimization methods is to achieve train energy savings, the specific principles and methods are different. The solution of the energy-saving optimization problem of the train is to find a series of parameters that meet the optimization objectives and constraints in the floating-point space, and it is necessary to take into account both the local search and global optimization at the same time to obtain the optimal solution. Because of the differences in principles and solving methods among the methods in the comparative experiment, the optimization results are different. 

The method proposed in this study is to replace the braking motion with the inertial motion of the train as far as possible to reduce the huge energy loss caused by the conversion efficiency and harmonics during the recovery and conversion of regenerative braking energy. The main principle of the method based on the utilization of regenerative braking energy is to feed the regenerative braking energy into the optimization goal of energy saving and recycle the regenerative braking energy to make up for the traction energy consumption. However, the recycling of regenerative braking energy is an extremely complex link, and regenerative braking energy needs to go through many links before it can enter the power grid. Energy storage equipment, energy conversion efficiency and harmonic components all have an important impact on the recovery and utilization of regenerative braking energy. In a word, there is still a lot of energy loss in the energy-saving optimization method based on the recycling of regenerative braking energy. However, it is a good choice to use regenerative braking instead of traditional braking to improve the service life of track facilities. The main principle of the energy-saving optimization method based on timetable optimization is to regard the whole train as a whole and achieve the effect of energy-saving operation through running-time optimization. However, the energy-saving optimization method based on timetable optimization does not fully consider the influence of train running line and speed, so this kind of method can be regarded as a macro energy-saving optimization method. If the energy-saving optimization methods such as operating lines and running speed are combined with the energy-saving optimization method based on timetable, it should be an ideal energy-saving optimization method. Further research work will be undertaken on this aspect in the future, and the comprehensive energy-saving optimization method of urban rail trains will be studied for multi-vehicle collaborative energy-saving optimization. 

In fact, the energy-saving optimization of the train is to find the operation parameter series or time series in the floating-point space to produce the minimum energy-consumption of the train. The solving process needs to balance local search and global optimization. For the solution based on the genetic algorithm, the local search ability is insufficient because the binary coding chromosome is used, and the solution space of the model is a large floating-point range. Although the chicken swarm optimization algorithm is a new solution method of the optimization problem, the global approximation ability is relatively strong. However, the chicken swarm optimization algorithm is difficult to implement because of too many parameters, and the corresponding solution vector pattern needs to be designed according to the characteristics of the optimization problem. Its local search ability depends on the solution vector pattern, which leads to the difficulty of the practical application. The solution method based on particle swarm optimization needs other auxiliary means to avoid falling into the local extremum problem. The particle swarm optimization algorithm is a good solution method of the optimization problem with floating-point space in the solution, and it is easy to implement parallel computing. The differential evolution algorithm is a relatively novel optimization problem-solving method, which can better balance local search ability and global search ability. It is an optimization method based on floating-point coding, which is very suitable for solving the energy-saving optimization problem of the train. The differential evolution algorithm needs few parameters, is simple and easy to implement, and can be well-combined with parallel computing systems to solve complex problems.

There are many studies on the optimization methods of train energy-saving, but the principles and solving methods are different. The main purpose of this study is to reduce the energy conversion loss as much as possible to meet the requirements of train operation regulations, and find an energy-saving optimization method that is easy to realize and engineer by replacing regenerative braking as far as possible. From the results of numerical experiments, the proposed method shows the ideal performance in the energy-saving optimization of the train. It assumes that the operation time of the Nanning Metro Line 1 in China is from 6:30 to 23:00. If the optimization results are extended to the whole line and the local electricity price is 0.7 RMB, it can save about 5 million RMB in one year after being optimized by the proposed method.

## 4. Discussion

The optimization problem of train traction energy-saving in urban rail transit is a multi-objective energy-saving optimization problem with complex constraints, so it is difficult to establish the optimization model directly through the train operation mechanism. Traction energy-saving optimization based on the regenerative braking energy utilization and traction energy-saving optimization based on the timetable are the two main research directions in this field. Due to the influence of the performance of the energy recovery equipment, the harmonic component of the regenerative braking energy and energy conversion efficiency, the regenerative braking energy has not been fully converted into the actual effective energy. The harmonic component of regenerative braking energy reduces the effective utilization rate of regenerative braking energy. The research on the optimization of the traction energy-saving based on the train timetable is mainly based on the optimization of the train operation schedule, but there is often a great difference between the model conditions and actual operation conditions, which results in optimization results that do not well solve the actual energy-saving optimization problem. 

The proposed method in this study is different from most of the previous research methods. It does not rely solely on the utilization of regenerative braking energy and the timetable. The purpose of this study is to realize the energy-saving optimization of the traction curve based on the combination of energy management optimization and train inertia motion, and to reduce unnecessary regenerative braking energy conversion and recovery links, so as to improve the actual energy utilization. An optimization model based on the combination of inertia motion and energy optimization is established by taking the maximum idle distance as the objective and the maximum allowable running speed, passenger comfort, train timetable, maximum allowable acceleration and kinematics equation as constraints. An adaptive dynamic multimodal differential evolution algorithm is proposed and a comprehensive method for solving multi-objective optimization problems based on the combination of the improved differential evolution algorithm and Q learning algorithm is applied to solve the energy-saving optimization model. Numerical experimental results show that the method is effective. The improved differential evolution algorithm proposed takes into account both the global convergence speed and local optimization accuracy; it effectively solves the optimization model of traction energy-saving and improves the convergence speed. This method is neither directly based on the regenerative braking energy feedback to the optimization objective function nor simply using the running schedule as the optimization objective function, but comprehensively considers the combination of the train inertia motion and energy optimization. The proposed method provides a new reference solution for the optimization problem of energy-saving in train operation.

From the numerical experimental results, although the train running time based on the proposed method is the largest, the whole running time is within the reasonable range allowed by the train schedule, and the proposed method can reduce the traction energy consumption as much as possible. Therefore, from the point of view of the train operation rules and actual energy-saving effect, the proposed method is an effective solution to the traction energy-saving optimization problem of the urban rail transit train.

## 5. Conclusions

In this study, an optimization model based on the combination of inertia motion and energy optimization is established by taking the maximum idle distance as the objective and, an adaptive dynamic multimodal differential evolution algorithm is proposed to solve the optimization model. The experimental results show that the proposed method presents important energy-saving characteristics and produces energy savings of about 11.2% under the same numerical experimental conditions. If the optimization results are extended to the whole Nanning Metro Line 1 in China and the local electricity price is 0.7 RMB, it can save about 6 million RMB in one year after being optimized by the proposed method. The established multi-objective energy-saving optimization model of the urban rail transit can fully consider many constraints in the process of train operation, which provides a new modeling reference for solving the problem of energy-saving optimization of urban rail transit. The proposed adaptive dynamic multimodal differential evolution algorithm has the ability to balance global optimization and local optimization, which provides a new reference solution for complex optimization problems. 

In the construction of the energy-saving optimization model, this study mainly considers how to increase the idle distance to reduce the energy consumption under other constraints. Although it does realize the energy-saving of the urban rail transit in this research work, there are many factors that affect the energy consumption of urban rail transit operation. There is still a need for schemes to optimize the factors or combination of factors to improve the energy-savings of urban rail transit. Exploring the multi-factor combination optimization method to realize the energy savings of urban rail transit will be the focus of the research work in the future. Although the adaptive dynamic multimodal differential evolution algorithm is successfully applied to solve the energy-saving optimization problem of urban rail transit with high computational complexity, the proposed adaptive dynamic multimodal differential evolution algorithm still has room for improvement in the hierarchical strategy. How to implement the adaptive threshold of the evolution degree of the optimal fitness value is the main research focus of our next study.

## Figures and Tables

**Table 1:** Main parameters of the simulation analysis.

Parameter Sequence Number	Parameter Symbol	Parameter Value
1	*c_1_*	0.0013
2	*c_2_*	0.014
3	*c_3_*	2.4
4	*c_4_*	600
5	*n*	55
6	*α*	0.15
7	*β*	0.1
8	*γ*	0.65
9	τ	0.1
10	*G*	228 t
11	*P*	90 t
12	*S*	2200 m
13	*a*_max_	1.2 m/s^2^
14	*v*_max_	80 km/h
15	*F*_Tmax_	330 kN
16	*T*	168 s
17	*CM*	0.5
18	*CR*	0.5

## Data Availability

Restrictions apply to the availability of these data. The data was obtained from Nanning Urban Rail Transit Company. It can be requested from the operation department by email with the permission of Nanning Urban Rail Transit Company.

## Notations

Symbol	Unit	Description
$w_{b}$	N/kN	Unit basic resistance
$c_{1}$		An empirical constant
$c_{2}$		An empirical constant
$c_{3}$		An empirical constant
v	km/h	Speed of the train
$w_{c}$	N/kN	Unit tunnel-additional resistance
L	m	Length of a tunnel
$w_{r}$	N/kN	Unit curve-additional resistance
Q		Constant
R	m	Radius of a curve
$w_{p}$	N/kN	Unit ramp-additional resistance
W	kN	Motion resistance of train
P	t	Mass of passengers, 1 t = 1000 kg
G	t	Empty mass of train, 1 t = 1000 kg
$F_{i}$		Inertial factor
g	N⋅m	Gravity acceleration
S	m	Distance between two stations
$\Delta s_{i}$	m	The length of the small segment
$\bar{w_{b}}$	N/kN	Average unit basic resistance
$\bar{W_{i}}$	kN	Average motion resistance
$\bar{v_{i}}$	km/h	Average speed of train in $\Delta s_{i}$
$E_{k}$	kJ	Kinetic energy of the train
M	t	Total mass of the train, 1 t = 1000 kg
p		Coefficient of rotary mass
$v_{is}$	km/h	Starting speed of train in $\Delta s_{i}$
$v_{ie}$	km/h	Terminal speed of train in $\Delta s_{i}$
$a_{i}$	$m/s^{2}$	Acceleration of train in $\Delta s_{i}$
$\Delta t_{i}$	s	Running time of train in $\Delta s_{i}$
$C_{i}$	kN	External resultant force on the train in sub-segment i
$F_{Ti}$	kN	Tractive force of the train in sub-segment i
$F_{T\max}$	kN	Maximum allowable tractive force of the train
$a_{\max}$	$m/s^{2}$	Maximum acceleration allowed
$v_{\max}$	km/h	Maximum speed allowed
α, β, γ and λ		Weight coefficients of the optimization objective
Φ		The whole optimization objective function
$F_{best}(k)$		The most adaptive value of the k generation
θ		The degree of change of the optimal fitness value
CM		The enhancement probability
SC		The enhancement factor
CR		The probability of replacement
$x_{ij}$		The j dimension of the i individual
$f_{b}$		Value of the basic fitness
$Z_{i}$		The penalty factor of violating the maximum acceleration limit
$V_{i}$		The penalty factor of violating the maximum speed limit
$A_{i}$		The penalty factor of violating the passenger comfort constraint
$T_{i}$		The penalty factor of violating the tractive force limit
ϑ		The control factor of passenger comfort, generally with a value less than 1.2
ε, v, ξ, δ and ρ		Constant
